# INDICES OF RESILIENCY IN CELLS FROM UM-HET3 MICE MAY CORRELATE WITH INDIVIDUAL FUTURE HEALTH OUTCOMES

**DOI:** 10.1093/geroni/igz038.372

**Published:** 2019-11-08

**Authors:** Kennedy S Mdaki, Yuhong Liu, Adam B Salmon

**Affiliations:** University of Texas Health at San Antonio, San Antonio, Texas, United States

## Abstract

The ability of an organism to respond to physical stresses and return to homeostasis (i.e. resilience) has been suggested to correlate with longevity. Here, we investigated whether this extends to resilience at a cellular level using primary fibroblasts isolated from tail skin of genetically heterogeneous young adult UM-HET3 mice. Cells isolated from each individual mouse (cell line) were tested in their response to concentrations of agents or conditions predicted to induce a cellular challenge, including paraquat, hydrogen peroxide, antimycin a, cadmium chloride, mdivi-1, thapsigargin, and nutrient starvation. Cell viability was monitored in real-time using an incucyte S3 live cell analysis system and we addressed the response following challenge as a marker of resilience. Cellular uptake of ethidium homodimer-1 was used to determine the loss of viability. Cellular bioenergetics were assessed using a seahorse XF24. We found that cell lines that were resistant to paraquat were also resistant to antimycin a, and hydrogen peroxide. Cell lines that were resistant to nutrient starvation were also resistant to mdivi-1. Indices of cellular bioenergetics status including ATP production rate and cell respiratory control ratio, revealed potential relationships with resiliency. Taken together, our data indicate that skin fibroblasts retain individual physiological programs that may in part explain the patterns of resiliency or sensitivity to a stressor at the organismal level. Since the cell lines tested in this study were obtained from living mice, future work will investigate whether these patterns of resiliency change with age and elucidate their utility in predicting future health outcome.

